# The Chernobyl Nuclear Catastrophe: Baverstock and Williams Respond

**Published:** 2007-05

**Authors:** Keith Baverstock, Dillwyn Williams

**Affiliations:** Department of Environmental Sciences, Faculty of Natural and Environmental Sciences, University of Kuopio, Kuopio, Finland, E-mail: Keith.baverstock@uku.fi; Strangeways Research Laboratory, Cambridge, United Kingdom

Nussbaum makes three points, namely that on the basis of the “source term” for the Chernobyl accident population, doses are underestimated by a factor up to 26, that Chernobyl dose estimates ignore exposure to alpha emissions, and that excess perinatal mortality and morbidity have been widely observed outside the main contaminated regions.

Nussbaum’s first point is pivotal because it provides the rationale for the claims that the health effects of the accident have been underestimated. We are not aware that the source term has been used in the estimation of doses. It was not for the most affected areas (Fairlie and Sumner 2006). As far as Europe is concerned, doses from isotopes of iodine and cesium have been estimated from surveys of ground deposition. As recently as 2006, the doses for all of Western Europe and much of Central and Eastern Europe were reestimated by Cardis et al. (2006); there is reasonable agreement between their estimates and those made within a few years of the accident [e.g., United Nations Scientific Committee on the Effects of Atomic Radiation (UNSCEAR) 1988]. We, therefore, do not accept that population beta and gamma doses from iodine and cesium have been grossly underestimated.

We do not dispute Nussbaum’s argument that additional doses may have been received from alpha emitters incorporated internally and that these might have been more than is acknowledged by UNSCEAR and the World Health Organization (WHO). Unfortunately the references quoted by Nussbaum as showing that at least 85% of the fuel was released are abstracts that provide no supportive evidence. It is generally accepted that about 3% of the nonvolatile elements present in the reactor were released; while these were mostly deposited close to the reactor, more distant contamination also occurred. We know of no reliable evidence that the majority of the nonvolatile elements were released, and it is accepted that a huge radioactive lava-like mass of fuel remains in the reactor. We are not qualified to comment on the nature of the explosion, but that is hardly an issue if the doses are correctly estimated.

We acknowledge that there have been ecologic studies of increased perinatal morbidity and mortality in areas where doses were low (i.e., of the order of a few millisieverts) (Korblein 2006), but other studies have found no effect (e.g., Dolk et al. 1999; Hausler et al. 1992). The much larger effects that would be expected in populations much closer to the accident, and thus more highly exposed, are not accepted by the WHO and International Atomic Energy Agency (IAEA), and small increases have been attributed to increased recording of minor abnormalities. This does not mean that there has been no effect, and that is one reason why we have called for a comprehensive health assessment of the accident (Williams and Baverstock 2006). These effects were not attributed to the Chernobyl accident by either Fairlie and Sumner (2006) or the Committee Examining Radiation Risks of Internal Emitters (2004).

It is undoubtedly the case that some have sought to downplay the importance of the health consequences of the accident, the WHO and the IAEA among them, but it is also true that others have sought to inflate the health consequences. Fairlie and Sumner (2006) rightly point out the uncertainties involved in reconstructing the accident and thus the need for value judgments in making health assessments. We have doubts about some of the claims made in Nussbaum’s letter, but by pointing out the discrepancies between the views of some scientists and the majority, he reinforces one of our main points. The continuing disputes over the consequences of the Chernobyl accident make it essential that a major international organization be created to support authoritative studies of the long-term effects of the world’s biggest nuclear accident.
